# Biologically Active Peptides from Corn Gluten Meal Improve Microbiota Disorders Caused by *Helicobacter pylori* Infection in Mice

**DOI:** 10.3390/molecules30030705

**Published:** 2025-02-05

**Authors:** Guanlong Li, Yongchao Xie, Quanxin Wang, Zhengfei Miao, Xiaolan Liu, Xiqun Zheng

**Affiliations:** 1 Heilongjiang Provincial Key Laboratory of Corn Deep Processing Theory and Technology, College of Food and Bioengineering, Qiqihar University, Qiqihar 161006, China; 03580@qqhru.edu.cn (G.L.); 2024916366@qqhru.edu.cn (Y.X.); 2023940915@qqhru.edu.cn (Q.W.); 03861@qqhru.edu.cn (Z.M.); 2 College of Food Science, Heilongjiang Bayi Agricultural University, Daqing 163319, China

**Keywords:** corn protein activity peptides, *Helicobacter pylori*, anti-inflammatory, intestinal microbiota, mouse model

## Abstract

This study investigated the potential effects of corn protein activity peptides (CPAPs) on inflammation response levels and gastrointestinal microbiota in *Helicobacter pylori* (*H. pylori*) infection mice. CPAPs significantly up-regulated the mRNA expression of the anti-inflammatory factor IL-10 and down-regulated the mRNA expression of the pro-inflammatory factors TGF-β, TLR4, MyD88, and NF-κB, indicating that CPAPs may antagonize *H. pylori*-induced inflammatory responses by inhibiting NF-κB signaling pathways. Through the intervention of CPAPs, *H. pylori* colonization in the stomach was significantly reduced. Additionally, the structural composition of the gastrointestinal microbiota improved, with an increase in abundance and diversity. These changes positively regulate gastrointestinal microbiota disorders in mice. In addition, the PICRUST function prediction of intestinal microbiota revealed that CPAPs may prevent or reduce metabolic disorders brought about by *H. pylori*, which improve biometabolic pathways by modulating intestinal microbiota composition. In conclusion, these findings suggest that CPAPs may prevent or mitigate metabolic disorders induced by *H. pylori*, offering theoretical support for the development of corn-protein-based functional foods.

## 1. Introduction

*Helicobacter pylori* (*H. pylori*) has gained increasing attention in recent years as a potent pathogen. The global prevalence of *H. pylori* infection varies greatly from country to country, with the current research showing that approximately 50% of the population is infected with *H. pylori* [1]. Studies have found a correlation between *H. pylori* infection and gastric diseases such as peptic ulcers, mucosal lymphoma, and stomach cancer [2]. The World Health Organization has reached a consensus on the dangers of *H. pylori* and classified it as a class I carcinogen [3]. For the eradication of *H. pylori*, triple and quadruple therapies are most commonly used. Antibiotic treatment regimens can also induce corresponding side effects such as antibiotic-induced diarrhea and gastrointestinal microbiota disorders [4]. In addition, antibiotic therapy can lead to resistance in *H. pylori*, resulting in less effective treatment and further health complications [5].

Many studies have found that *H. pylori* infection also changes the structural composition of the gastrointestinal microbiota and triggers the microecological balance of the gastrointestinal microbiota, which not only affects nutrient absorption but also leads to a variety of related diseases [6]. Some non-antibiotic food-active substances from natural sources, such as polyphenols from cranberries [7], peptides [8], and water-soluble polysaccharides from okra [9], have been shown to antagonize *H. pylori* infection, which mainly inhibits *H. pylori* adhesion and colonization, and reduce gastric inflammation, but the effects of these substances on the gastrointestinal microbiota are unknown as of yet.

A large number of corn polypeptides can be found through hydrolytic corn proteins in proper condition. These peptides are easy to take in and have various biological functions such as showing antioxidant activity, promoting ethanol metabolism, and being antihypertensive and hypoglycemic [10,11,12,13]. In our previous study, we found that corn-protein-derived peptides (CPAPs) could significantly inhibit *H. pylori* adhesion to GES-1 cells in vitro and could reduce the colonization of *H. pylori* in the stomach in mice and alleviate the inflammatory response in vivo [14]. However, it is not clear whether they have a regulating effect on the gastrointestinal microbiota disorder caused by *H. pylori* infection. The intervention with active peptides has been found to positively impact the structure of the gut microbiota [15,16,17]. Therefore, we speculated that CPAPs could improve gastrointestinal microbiota disorders induced by *H. pylori* infection.

In this study, CPAPs were gavaged to intervene in *H. pylori* infection and we analyzed the mRNA expression of inflammatory factors in the gastric mucosa. The regulatory effects of CPAPs on gastrointestinal microbiota disorders were systematically analyzed by 16S rRNA sequencing. This study uncovered novel functions of CPAPs, laying the groundwork for the development of related functional foods.

## 2. Results and Discussion

### 2.1. Effects of CPAP Intervention on mRNA Expression Levels of Inflammatory Factors in Gastric Mucosal

*Helicobacter pylori* adheres to the gastric mucosa, releasing virulence factors that cause gastric mucosal damage [14]. In order to further analyze the antagonistic effect of CPAPs on gastric mucosal damage induced by *H. pylori* infection in mice, the mRNA expression levels of five key inflammatory factors were assayed.

**Figure 1:**
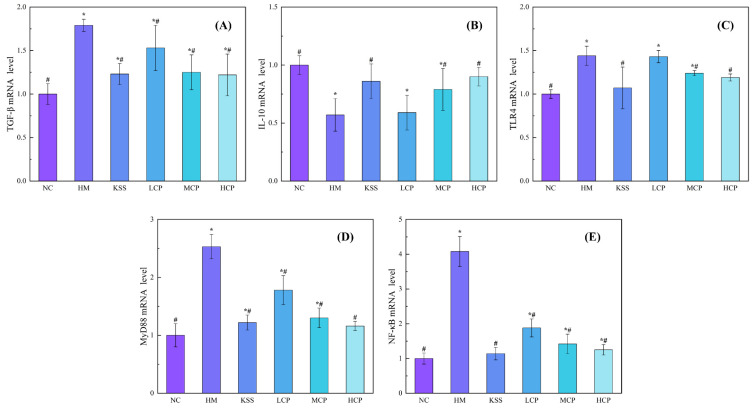
CPAPs modulate the inflammatory cytokine mRNA expression level of gastric mucosa. (**A**) Transforming growth factor-β (TGF-β) mRNA expression levels; (**B**) Interleukin-10 (IL-10) mRNA expression levels; (**C**) Toll-like receptor 4 (TLR4) mRNA expression levels; (**D**) Myeloid differential protein88 (MyD88) mRNA expression levels; (**E**) Nuclear transcription factor-κB (NF-κB) mRNA expression levels. * denotes significant difference from NC group (*p* < 0.05); # denotes significant difference from HM group (*p* < 0.05). NC group: normal control group, HM group: *H. pylori* model group, KSS group: positive group, LCP group: CPAPs with low dose (200 mg/kg·bw), MCP group: CPAPs with medium dose (400 mg/kg·bw), and HCP group: CPAPs with high dose (600 mg/kg·bw).

Transforming growth factor-β (TGF-β) is an important immunomodulatory factor in the body that can inhibit the proliferation and differentiation of immunoreactive cells in the body and thus affect the production of cytokines and antibodies [18]. Studies had revealed a significant increase in TGF-β expression in the gastric tissues of patients infected with *H. pylori* [3]. However, following treatment, the expression of TGF-β in these tissues decreased. In addition, the expression of TGF-β is also related to the development of gastric cancer [19]. As shown in Figure 1A, compared with the normal control group (NC), the mRNA expression level of TGF-β factor was significantly up-regulated in the *H. pylori* model group (HM) (*p* < 0.05), indicating that *H. pylori* infection was able to stimulate the gastric mucosal epithelial cells, inducing an increase in the secretion of TGF-β and resulting in the local immune deficiency in the gastric mucosa, which led *H. pylori* to evade the immune clearance reaction and gain the opportunity to survive in vivo. After CPAP intervention, with an increase in the CPAP intervention dose, the TGF-β mRNA expression level in the gastric mucosa was significantly down-regulated (*p* < 0.05), and there was no significant difference compared with the normal group (*p* > 0.05). These results showed that the intervention of CPAPs could significantly down-regulate the mRNA expression of TGF-β factor in the gastric mucosa, increase the immune clearance function against *H. pylori*, reduce the inflammatory response, and alleviate the damage response of gastric mucosal.

Interleukin-10 (IL-10) is an intracellular anti-inflammatory factor that can inhibit the production of inflammatory factors and thus inhibit the inflammatory response. As shown in Figure 1B, compared with the NC group, the mRNA expression level of IL-10 in the gastric mucosa of the HM group was significantly down-regulated (*p* < 0.05), indicating that the *H. pylori* infection increased the inflammatory response, which led to a decrease in the expression of the anti-inflammatory factor, which was consistent with the results reported in the literature [20]. Compared with the HM group, IL-10 expression in gastric tissues was significantly up-regulated after CPAP intervention (*p* < 0.05) and showed a dose-dependent effect with an increase in the CPAP dose. Under the intervention of CPAPs at a dose of 600 mg/kg·bw, there was no significant difference in IL-10 expression level compared with the NC group. The results showed that CPAP intervention could significantly up-regulate IL-10 mRNA expression levels (*p* < 0.05) and then inhibit the occurrence of the gastric mucosal inflammatory response.

The pattern recognition receptor Toll-like receptor 4 (TLR4) present on the surfaces of gastric mucosal epithelial cells can specifically recognize the virulence factors LPS and CagA, which are released by *H. pylori* [21]. The complex formed by the combination of the two can activate the immune response of the body, which in turn promotes the increase in the aggregation of the key regulatory molecule myeloid differential protein88 (MyD88) and the formation of the TLR4/MyD88 complex. Through the activation of the nuclear transcription factor-κB (NF-κB) signaling pathway cascade, it promotes an excessive increase in the NF-κB content and contributes to the inflammatory response of the gastric mucosal [22]. The results of the effects of CPAPs on the expression of TLR4, MyD88, and NF-κB in gastric mucosa are shown in Figure 1C–E. Compared with the NC group, the mRNA expression levels of TLR4, MyD88, and NF-κB of the HM group were significantly up-regulated (*p* < 0.05), and these were 1.44, 2.53, and 4.08 times higher than those of the NC group, respectively, indicating that the *H. pylori* infection induced an increase in the mRNA expression level of TLR4 in the gastric mucosa, which led to an increase in the expression of MyD88 and NF-κB, activating the NF-κB signaling pathway, resulting in active inflammatory factor anabolism. Tong et al. also found that the NF-κB signaling pathway was activated via *H. pylori* infection, leading to an increase in the content of TLR4 and MyD88 [23]. Compared with the HM group, CPAP intervention could significantly down-regulate the mRNA expression levels of TLR4, MyD88, and NF-κB (*p* < 0.05), and the mRNA expression levels of TLR4, MyD88, and NF-κB in the CPAP group were gradually reduced with an increase in the CPAP intervention dose. Among them, there were no significant differences in TLR4, MyD88, and NF-κB expression levels in the high-dose group compared with those in the NC group (*p* > 0.05). These results indicated that CPAP intervention could reduce TLR4 production by decreasing the amount of *H. pylori* colonization in the stomach and then inhibit the increases in the expressions of MyD88 and NF-κB, inhibiting the activation of the NF-κB signaling pathway and alleviating the *H. pylori*-infection-induced gastric mucosal damage.

Based on our previous study [14], CPAPs inhibit *H. pylori* colonization in the gastric mucosa. This protective effect likely occurs through multiple mechanisms: inhibiting *H. pylori* adhesion, reducing TGF-β expression, and enhancing gastric mucosal immune function, thereby increasing *H. pylori* clearance. In addition, by inhibiting the formation of the TLR4/MyD88 complex, which in turn inhibits the activation of the NF-κB signaling pathway, it down-regulates the content of pro-inflammatory factors and up-regulates the content of anti-inflammatory factors, which alleviates the gastric mucosal damage response caused by *H. pylori*.

### 2.2. Regulatory Effect of CPAPs on Gastric Microbiota Disorders by H. pylori Infection

Several studies have indicated that *H. pylori* infection can lead to disruptions in the gastrointestinal microbiota, and these disturbances are closely linked to the progression of various diseases [24]. Previous experimental research has demonstrated that CPAPs are capable of reducing *H. pylori* colonization in the stomach, subsequently alleviating oxidative stress and inflammatory responses in the gastric mucosa [14]. Nevertheless, the specific regulatory effect of CPAPs on gastrointestinal microbiota imbalances resulting from *H. pylori* infection remains unexplored. Consequently, this study aimed to investigate the role of CPAPs in regulating gastrointestinal microbiota disorders. In addition, in this study, we mainly wanted to explore the regulatory effect of CPAP on gastrointestinal microflora disturbance induced by *H. pylori* infection. Under normal circumstances, *H. pylori* infection causes the disruption of the gastrointestinal microflora. At present, triple antibiotic therapy is mainly used in the clinical treatment of *H. pylori* infection, so we chose antibiotics as a positive control. This comparison could also compare the effects of active peptides and antibiotics on gastrointestinal microflora.

#### 2.2.1. Species Diversity Correlation Curve Analysis of the Gastric Microbiota

**Figure 2:**
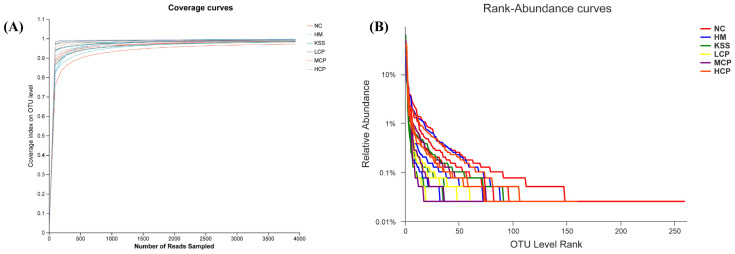
Analysis of species diversity correlation curve of the gastric microbiota. (**A**) Dilution curve: the dilution curve is mainly used to evaluate the abundance of species in the samples. (**B**) Rank-Abundance curve: a Rank-Abundance curve is a way to analyze the diversity of a sample. NC group: normal control group, HM group: *H. pylori* model group, KSS group: positive group, LCP group: CPAPs with low dose (200 mg/kg·bw), MCP group: CPAPs with medium dose (400 mg/kg·bw), and HCP group: CPAPs with high dose (600 mg/kg·bw).

The dilution curve serves primarily to assess species abundance in samples, facilitating comparisons of species diversity across varying amounts of sequencing data. Additionally, it provides an indication of the adequacy of the sequencing data volume for the samples [25]. The steepness on the curve’s left side signifies a higher diversity of species identified through sequencing. As the curve flattens, it suggests that adequate sequencing data have been obtained, indicating a reasonable data volume. Further data mining is likely to yield only a limited number of new OTUs. Conversely, if the curve remains uneven, it suggests the potential for discovering more new OTUs through continued sequencing. As illustrated in Figure 2A, with an increasing sequencing volume, the dilution curves for different sample groups gradually flatten, indicating broader sequencing depth and richer species coverage. This demonstrates the reasonableness and reliability of the sequencing results, accurately reflecting the samples’ actual state and suitability for further analysis and research.

The Rank-Abundance curve serves as a method to assess sample diversity. It involves sorting the OTUs within the sample based on their relative abundance, from highest to lowest, obtaining a sorting number, and plotting the curve using this number as the horizontal coordinate and the relative percentage of sequences within each OTU as the vertical coordinate [26]. The Rank-Abundance curve provides a visual representation of species abundance and uniformity within the samples. The width of the curve indicates species richness while its smoothness reflects the evenness of species distribution. With an increase in the number of stomach microorganisms, their relative abundance gradually declined (Figure 2B). When the relative abundance in the samples dropped below 0.1% in the study, the curves became straighter and entered a plateau phase, indicating a higher diversity of microorganisms in the mouse stomach contents. Meanwhile, the curve reached the plateau period more gently, indicating better microbial homogeneity. The highest species abundance was found in the NC group and the lowest in the KSS and HM groups, indicating that both *H. pylori* infection and antibiotic intake decreased the species abundance of microorganisms in the stomach and that the microbial species abundance could be regulated by a certain dose of CPAP intervention. The overall trend of the samples in each group was flat, indicating a good species distribution uniformity.

#### 2.2.2. OTUs and Community Composition Analysis of the Gastric Microbiota

**Figure 3:**
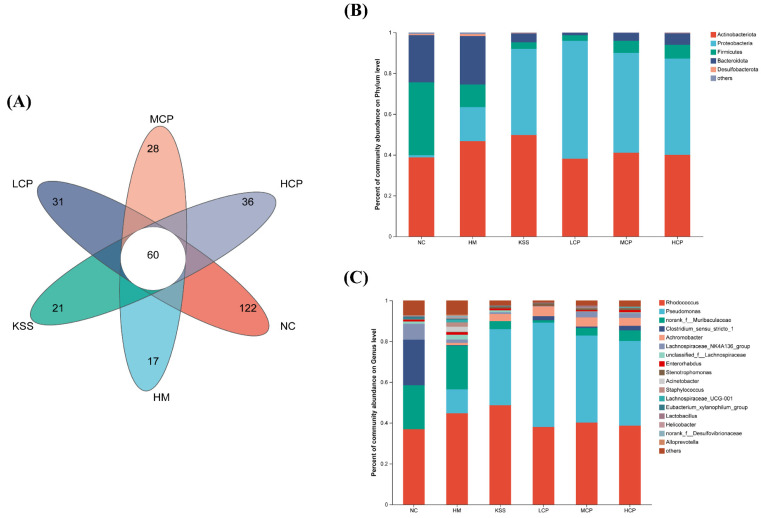
OTUs and community composition analysis of the gastric microbiota. (**A**) Venn diagrams. The relative abundance of the gastric microbiota at phylum level (**B**) and genus level (**C**). NC group: normal control group, HM group: *H. pylori* model group, KSS group: positive group, LCP group: CPAPs with low dose (200 mg/kg·bw), MCP group: CPAPs with medium dose (400 mg/kg·bw), and HCP group: CPAPs with high dose (600 mg/kg·bw).

A Venn diagram was obtained by the comparative analysis of OTUs between group samples. The Venn diagram illustrates the number of shared and unique OTUs among different sample groups, and it was created after homogenizing all samples. As shown in Figure 3A, there were variations in the numbers and types of OTUs among the samples of each group. The total number of OTUs across all groups amounted to 60. Specifically, the NC, HM, KSS, LCP, MCP, and HCP groups uniquely possessed 122, 17, 21, 31, 28, and 36 OTUs, respectively. The subjects in the NC group exhibited the highest number of OTUs in their stomachs whereas the HM and KSS groups showed the lowest, aligning with the findings from the species diversity correlation curve. These results indicated that after *H. pylori* infection, the species composition of stomach microorganisms in the HM group changed significantly compared to the NC group, and the number of OTUs was significantly reduced. The results are consistent with what Yang et al. reported [27]. Compared with the HM group, CPAP intervention effectively alleviated the reduction in the number of gastric microbial OTUs, with the highest number of OTUs being in the HCP group, which was still significantly different from the NC group. In addition, antibiotic intake was also able to significantly reduce the number of OTUs of the gastric microorganisms in mice. The results indicated that the reduction in the number of OTUs of the gastric microorganisms in mice could be alleviated by a certain dose of CPAP intervention.

The community composition of gastric microbiota was analyzed at the phylum level. As shown in Figure 3B, there were five phyla with relative abundance above 1%, namely *Actinobacteriota*, *Proteobacteria*, *Firmicutes*, *Bacteroidota*, and *Desulfobacteria*. *Firmicutes* and *Bacteroidota* are the dominant beneficial bacteria in the body, and changes in their abundance have been associated with the pathogenesis of obesity, gastrointestinal cancers, diabetes, and other diseases [28]. *Firmicutes* species mainly take proteins and carbohydrates as nutrients and hydrolyze the two to produce butyric acid, which inhibits the proliferation of intestinal cancer cells [29]. *Bacteroidota* mainly takes polysaccharides, bile acids, and steroids as the main nutrients and generates propionic acid, acetic acid, and other short-chain fatty acids for nutrient absorption. At the same time, these short-chain fatty acids can also inhibit the TNF-α release [30]. *Proteobacteria* mainly include a variety of pathogenic bacteria that are prone to gastrointestinal disease [28]. In addition, it was found that the number of *Firmicutes* specimens was significantly up-regulated and the number of *Bacteroidota* specimens was significantly down-regulated in the obese population [31]. Significant changes in the relative abundance of *Firmicutes* were observed in the HM group compared to the NC group, with a 25.70% decrease in the relative abundance of *Firmicutes* and no significant change in the relative abundance of *Bacteroidota*, suggesting that infection with *H. pylori* is capable of significantly altering the species composition of the gastric microbiota at the phylum level, specifically through a decrease in the number of beneficial bacteria and an increase in the number of harmful bacteria. Compared with the HM group, the gastric microbiota disorders caused by *H. pylori* infection could be improved, and the structure of the microbiota could be regulated by the intervention of different doses of CPAPs, so that the composition of the microbiota tended to the NC group. The community structure of the gastric microbiota in the KSS group was also significantly different from that of the NC group, which showed that the intake of antibiotics still leads to the structural disorganization of the gastric microbiota.

The genus is the smallest unit that can be accurately recognized in 16S sequencing and is the unit in the body that has its regulatory role. Therefore, the community composition of the gastric microbiota was analyzed at the genus level. As shown in Figure 3C, there were 17 genera with a relative abundance greater than 1%, and the main dominant bacteria at the genus level included *g_Rhodococcus*, *g_Pseudomonas*, and *norank_f_Muribaculacea*. Compared with the NC group, the content of *Clostridium_sensu_stricto_1*, *Pseudomonas*, and *Helicobacter* in the HM group was significantly decreased, indicating that *H. pylori* infection led to the disorganization of the gastric microbiota structure in the mice, and *g_Pseudomonas*, *g_Helicobacter*, and other harmful bacteria increased, which was consistent with previous studies reported [29], in which increases in the levels of *g_Helicobacter* in the stomach were directly related to the modeling gavage *H. pylori*. Compared with the HM group, the content of *g_Rhodococcus* and *g_Helicobacter* was significantly reduced by CPAP intervention. CPAP intervention inhibited the colonization of *H. pylori* in the stomach, which was manifested by a significant reduction in the number of *Helicobacter* genera in the stomach. Saresella et al. found a positive correlation between the number of *Lachnospiraceae_NK4A136_group* specimens and the number of anti-inflammatory immune cells in the body [32]. Compared with the NC group, the *Lachnospiraceae_NK4A136_group* content in the HM group was significantly reduced. In contrast, the *Lachnospiraceae_NK4A136_group* was significantly increased in the CPAP groups (*p* < 0.05), and the body’s anti-inflammatory level was increased. This was consistent with the elevated anti-inflammatory factors in the CPAP-group gastric mucosa and also consistent with our previous study [14]. Based on the genus level, gastric microbiota community analysis showed that CPAPs could inhibit the adhesion and colonization of *H. pylori*, reducing *H. pylori* levels in the stomach, which was consistent with our previous in vitro study. In summary, the CPAP intervention can improve the structure of gastric microbiota community composition, increase the number of beneficial bacteria and reduce the number of harmful bacteria, and improve the structural disorder of gastric microbiota caused by *H. pylori* infection.

#### 2.2.3. Microbiota Diversity Analysis of the Gastric Microbiota

**Figure 4:**
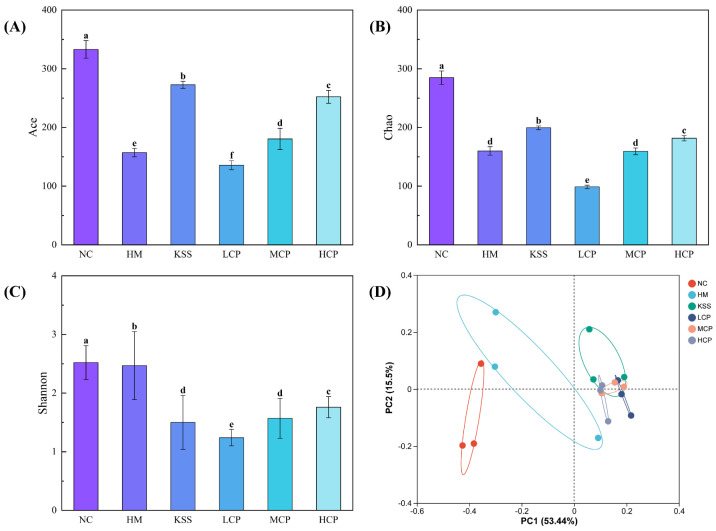
Microbiota diversity analysis of the gastric microbiota. (**A**) Ace index; (**B**) Chao index; (**C**) Shannon index; (**D**) PCoA analysis. Different lowercase letters represent significant differences (*p* < 0.05). NC group: normal control group, HM group: *H. pylori* model group, KSS group: positive group, LCP group: CPAPs with low dose (200 mg/kg·bw), MCP group: CPAPs with medium dose (400 mg/kg·bw), and HCP group: CPAPs with high dose (600 mg/kg·bw).

The Shannon index is used to reflect the biodiversity of microbial species. The Ace index is mainly used to estimate the number of OTUs contained, and it is often used to estimate the total number of OTUs in samples. The Chao index is an algorithm that uses the chao1 algorithm to estimate the number of OTUs contained in a sample, and it is often used to estimate the total number of species in samples. Unlike the Ace algorithm, the larger the Chao index, the greater the number of OTUs, indicating a higher species richness in the samples [33]. Compared with the NC group, the richness indices Ace and Chao, and the diversity index Shannon, were significantly decreased in the HM group (*p* < 0.05), indicating that *H. pylori* infection caused a substantial decrease in species richness and microbial diversity in the gastric microbiota, leading to structural disorders (Figure 4A–C). Compared with the HM group, the diversity index Shannon, richness index Ace, and Chao index increased after intervention with different doses of CPAPs, and the indices showed a dose-dependent significant increase with an increase in the CPAP dose (*p* < 0.05). The high dose of CPAP intervention could not restore the species richness and microbial diversity to the normal level, but it was close to that of the NC group. This indicates that CPAP intervention can significantly restore the species richness and microbial diversity of gastric microbiota and regulate the structure. Compared with the HM group, the KSS group showed a decrease in the diversity index Shannon and an increase in the richness indices Ace and Chao, indicating that antibiotic intake could reduce microbial diversity and significantly increase the richness within the gastric microbiota.

Principal Coordinate Analysis (PCoA) is a method to analyze the similarity of or difference between research data through visualization. After sorting through a series of eigenvectors and eigenvalues and selecting the eigenvalues that are mainly in the top rankings, differences between different sample groups can be observed through PCoA. As shown in Figure 4D, the cumulative variance contributions of the first two principal components were 53.44% and 15.50%, respectively. The HM and NC groups were separated, indicating significant differences in species composition between the two groups. The CPAPs and the KSS group were clearly demarcated from the HM group, suggesting that the microbial community structure was clearly differentiated between groups. LCP, MCP, and HCP had intersecting sections, representing a gradual increase in community composition with an increasing CPAP dose. In conclusion, based on the analysis of the β-diversity results of gastric flora, CPAP intervention can significantly regulate the microbiota diversity in a positive direction.

#### 2.2.4. Microbial Community Biomarker Analysis Based on LEfSe

**Figure 5:**
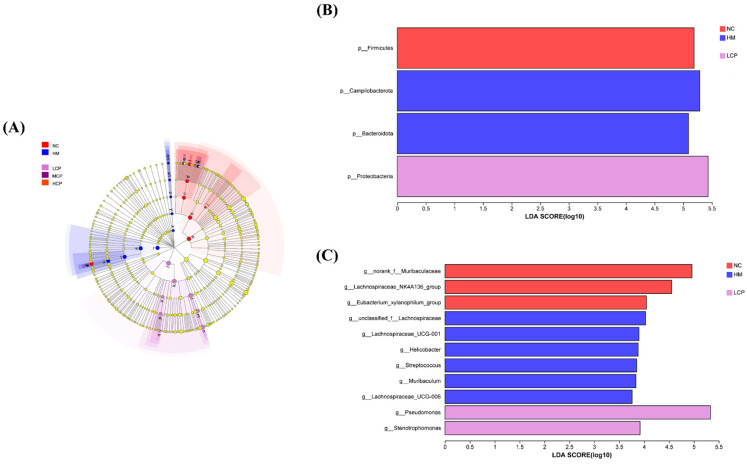
Analysis of evolutionary branching based on LEfSe and the LDA score was 3.0 (**A**) at phylum level (**B**) and genus level (**C**) of the gastric microbiota. LEfSe was used to identify the enrichment and changes in microbial communities of various populations between groups. NC group: normal control group, HM group: *H. pylori* model group, KSS group: positive group, LCP group: CPAPs with low dose (200 mg/kg·bw), MCP group: CPAPs with medium dose (400 mg/kg·bw), and HCP group: CPAPs with high dose (600 mg/kg·bw).

To further analyze biomarkers with significant differences in relative abundance between groups, LEfSe was used to identify the enrichment and changes in the microbial communities of various populations between groups. As shown in Figure 5, the total number of taxonomic classes with LAD values above 2.0 was 28. At the phylum level, *Firmicutes* was significantly enriched in the NC group (LDA > 5.0), *Campilobacterota* and *Bacteroidota* were significantly enriched in the HM group (LDA > 5.0), and *Proteobacteria* were significantly enriched in the LCP group (LDA > 5.0).

At the genus level, the HM group had the most characteristic communities, the NC group had the second most, the LCP group had the least characteristic communities, and the remaining groups had no characteristic communities. The NC groups *norank_f_Muribaculaceae*, *Lachnospiraceae_NK4A136_group*, and *Eubacterium_xylanophilum_group* were significantly enriched (LDA > 4.0), and the HM groups *unclassified_f_Lachnospiraceae* (LDA > 4.0), *Lachnospiraceae_UCG-001*, *g*_*Helicobacter*, *g_Streptococcus*, *g_Muribaculum*, and *Lachnospiraceae_UCG-006* were significantly enriched (LDA > 3.5). *Pseudomonas* (LDA > 5.0) and *Stenotrophomonas* (LDA > 3.5) were significantly enriched in the LCP group. The above communities can be considered as potential biomarkers for each group. The abundance of *g_Helicobacter*, *g_Streptococcus*, *g_Muribaculum*, *unclassified_f_Lachnospiraceae*, and *Lachnospiraceae_UCG-006* in the HM group was significantly higher than that in the NC group, and it is hypothesized that the high abundance of these microorganisms is directly related to *H. pylori* infection. Some studies had also found that *g_Helicobacter* and *g_Muribaculum* were important markers by the model infection group, which was consistent with the present study results [24,26]. In addition, *g*_*Helicobacter* was one of the potential markers in the HM group, which was consistent with the fact that the HM group was infected, resulting in an increase in the number of *H. pylori* specimens. *g*_*Helicobacter* was not seen in the remaining groups, further suggesting that CPAP intervention had significantly reduced *H. pylori* colonization. Taken together, the results analyzed above indicate that CPAP intervention had a positive effect on *H. pylori*-induced gastric microbiota dysbiosis.

### 2.3. Regulatory Effect of CPAPs on Intestinal Microbiota Disorders by H. pylori Infection

#### 2.3.1. Species Diversity Correlation Curve Analysis of the Intestinal Microbiota

**Figure 6:**
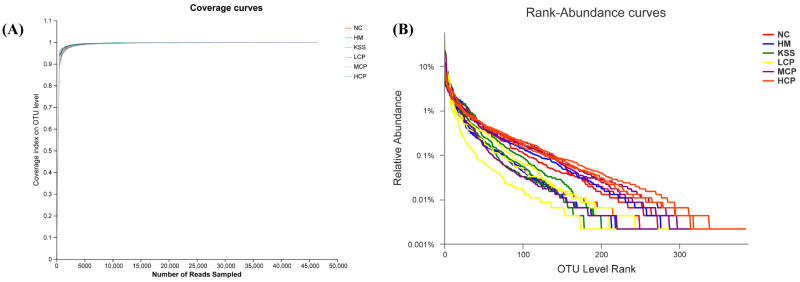
Analysis of species diversity correlation curve of the intestinal microbiota. (**A**) Dilution curve: the dilution curve is mainly used to evaluate the abundance of species in the samples. (**B**) Rank-Abundance curve: a Rank-Abundance curve is a way to analyze the diversity of a sample. NC group: normal control group, HM group: *H. pylori* model group, KSS group: positive group, LCP group: CPAPs with low dose (200 mg/kg·bw), MCP group: CPAPs with medium dose (400 mg/kg·bw), and HCP group: CPAPs with high dose (600 mg/kg·bw).

The gastric microbiota has been studied more extensively owing to *H. pylori* colonizing the gastric mucosa. However, disturbances in the gastric microbiota also affect the intestinal microbiota, and the relationship between intestinal microbiota and disease development is more important. It has been found that *H. pylori* infection is not only able to cause disorders of gastric microbiota but also has an important effect on the structural composition of intestinal microbiota [34,35]. Therefore, we further analyzed the regulatory effect of CPAPs on intestinal microbiota disorders by *H. pylori* infection. As shown in Figure 6, as the number of intestinal microbiota in each group increased, their relative abundance gradually decreased. When the relative abundance in the samples was lower than 0.1%, the curve tended to be straight and entered into the plateau period, and most of the samples contained pairs of OTUs in the range of 200 to 300, indicating that the microbial abundance in the intestinal contents of mice in each group was high. At the same time, the curve arrived at the plateau period in a relatively flat manner, indicating that microbial uniformity was better. The highest species richness was found in the HCP group while the lowest was found in the KSS and HM groups, suggesting that *H. pylori* infection and antibiotic intake could reduce the species abundance of microorganisms in the intestine and that intervention with a certain dose of CPAPs could regulate the abundance of microorganisms in the intestine.

#### 2.3.2. OTUs and Community Composition Analysis of the Intestinal Microbiota

**Figure 7:**
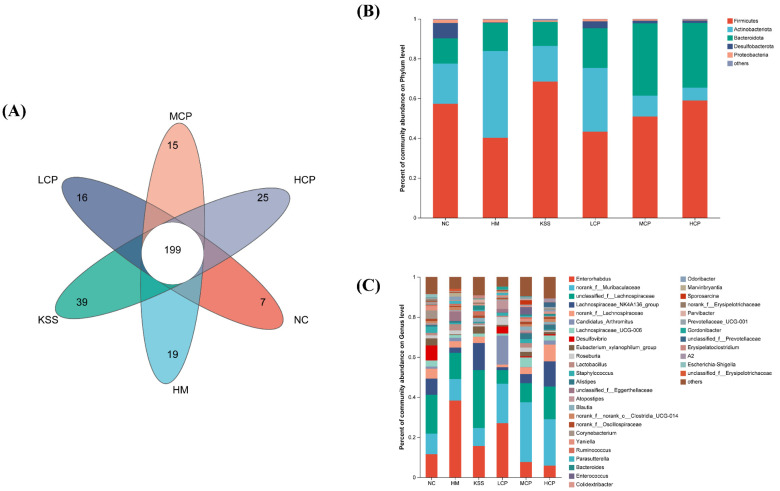
Venn diagrams analysis of the intestinal microbiota (**A**). The relative abundance of the intestinal microbiota at phylum level (**B**) and genus level (**C**). NC group: normal control group, HM group: *H. pylori* model group, KSS group: positive group, LCP group: CPAPs with low dose (200 mg/kg·bw), MCP group: CPAPs with medium dose (400 mg/kg·bw), and HCP group: CPAPs with high dose (600 mg/kg·bw).

As shown in Figure 7A, there were differences between the numbers and types of OTUs in the samples of each group. The total number of OTUs in all groups was 199, and the NC, HM, KSS, LCP, MCP, and HCP groups uniquely had OTUs of 7, 19, 39, 16, 15, and 25 each, respectively, suggesting that after the mice were infected by *H. pylori*, the species composition of intestinal microorganisms in the HM group was significantly changed compared to that in the NC group. Compared with the HM group, the number of unique OTUs in the LCP and MCP groups decreased significantly, and the number of unique OTUs in the HCP increased slightly, indicating that a certain dose of CPAP intervention could significantly change the species composition of intestinal microorganisms and that the intake of antibiotics could significantly change the structures of intestinal microorganisms in mice.

At the phylum level, there were five phyla with relative abundance above 1%, and the dominant phylum species were the same in each group, but the compositions differed among the groups. Compared with the NC group, the relative abundance of *Firmicutes* and *Actinobacteriota* in the HM group underwent significant changes, in which the relative abundance of *Firmicutes* decreased by 29.96% and the relative abundance of *Actinobacteriota* increased by 23.30%. There was no significant change in the relative abundance of *Bacteroidota*, suggesting that *H. pylori* infection can significantly change the species composition of intestinal microbiota in mice, resulting in decreases in the number of beneficial bacteria and increases in the number of harmful bacteria. This finding was consistent with previous reports in the literature [36,37]. Meanwhile, the ratio of *Firmicutes*/*Bacteroidota* in the HM group was significantly reduced by 37.48%, indicating that *H. pylori* infection may lead to a disproportionate ratio of *Firmicutes* and *Bacteroidota* in the intestine. Compared with the HM group, the relative abundance of *Firmicutes* and *Bacteroidota* in each dose group increased with increasing doses of CPAP intervention by 7.53%, 27.52%, and 45.00% and 26.30%, 76.09%, and 84.13%, respectively. The relative abundance of *Actinobacteriota* and *Proteobacteria* showed a significant decrease, for which the HCP group had the best effect, and the relative abundance of various bacterial phyla was basically restored to the normal level, which indicated that the CPAP intervention could effectively improve the structural disorder of the intestinal microbiota induced by the infection of *H. pylori*.

From the genus-level analysis, there were 37 genera with a relative abundance greater than 1% in mice’s intestinal microbiota, and there were three dominant organisms mainly by the genus level, *Enterorhabdus*, *norank_f_Muribaculaceae*, and *unclassified_f_Lachnospiraceae*. The genus *Enterobacteriaceae* includes *Shigella*, *Salmonella*, *Klebsiella*, *Rubella*, and *Enterobacteriaceae*, most of which belong to the genus of pathogenic bacteria [38,39]. *Lachnospiraceae* is a probiotic bacterium in the intestinal that ferments glucose to produce formic acid, lactic acid, acetic acid, and ethanol [40,41]. Compared with the NC group, the relative abundance of *Enterorhabdus* in the HM group increased significantly, and the relative abundance of *Lachnospiraceae* and *norank_f_Muribaculaceae* decreased, indicating that *H. pylori* infection resulted in the disorganization of the intestinal microbiota, the relative abundance of beneficial bacteria such as *g_Lachnospiraceae* decreased, and the relative abundance of pathogenic bacteria *g_Enterorhabdus* increased substantially. Compared with the HM group, the relative abundance of *g_Enterorhabdus* in the CPAP group was significantly reduced by 29.32%, 79.84% and 84.81%. Several studies have found that food-borne actives can improve the gastrointestinal microbiota induced by *H. pylori* infection by modulating the microbial community structure [42,43,44]. Meanwhile, the results indicated that the CPAP intervention would significantly improve the relative abundance of different genera induced by *H. pylori* infection, inhibit the proliferation of pathogenic bacteria, and stabilize the species diversity of intestinal microbiota and that its regulatory effect was related to its dose.

#### 2.3.3. Microbiota Divers

**Figure 8:**
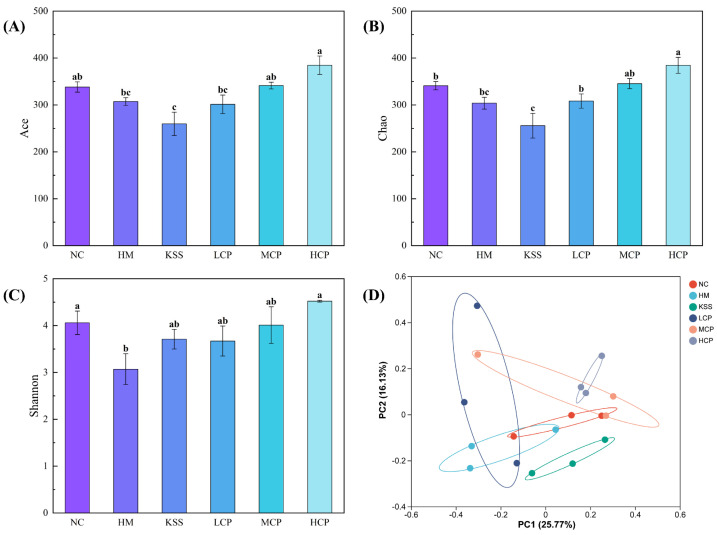
Microbiota diversity analysis of the intestinal microbiota. (**A**) Ace index; (**B**) Chao index; (**C**) Shannon index; (**D**) PCoA analysis. Different lowercase letters represent significant differences (*p* < 0.05). NC group: normal control group, HM group: *H. pylori* model group, KSS group: positive group, LCP group: CPAPs with low dose (200 mg/kg·bw), MCP group: CPAPs with medium dose (400 mg/kg·bw), and HCP group: CPAPs with high dose (600 mg/kg·bw).

Compared with the NC group, the richness indices Ace and Chao, and the diversity index Shannon, were significantly decreased in the HM group (*p* < 0.05), indicating that the species richness and microbial diversity in the intestinal microbiota were substantially reduced after *H. pylori* infection, leading to structural disorders of the intestinal microbiota. As shown in Figure 8, compared with the HM group, the diversity index Shannon and richness indices Ace and Chao increased after intervention by different doses of CPAPs, and the indices showed a dose-dependent significant increase with increases in the CPAP dose (*p* < 0.05). The Shannon, Ace, and Chao indices could be restored to the normal level in the MCP group, and the levels of the three indices in the HCP group were higher than those in the NC group, but there was no significant difference, which indicated that the CPAP intervention could significantly restore the species richness and microbial diversity of the intestinal microbiota. Compared with the HM group, the diversity index Shannon of the KSS group was significantly higher, and the richness indices Ace and Chao were significantly lower than those of the HM group, indicating that antibiotic intake could increase the microbial diversity within the intestinal microbiota of mice, and the richness was significantly reduced *(p* > 0.05). The improvement of microbial community diversity in the gastrointestinal tract may be one of the mechanisms of CPAP action.

As shown in Figure 8D, the cumulative variance contributions of the first two principal components were 25.77% and 16.13%, respectively. The HM and NC groups were clearly separated, indicating significant differences in species composition. Compared with the distance between the NC and HM groups, the distance between the CPAP (LCP, MCP, and HCP) and NC groups was significantly shorter, indicating that the structure of the intestinal microbiota composition tended to the NC group by CPAP intervention. Among them, the MCP and HCP groups showed the best effect, which was closer to the NC group, while the LCP and KSS groups showed some differences.

#### 2.3.4. Microbial Community Biomarker Analysis Based on LEfSe of the Intestinal Microbiota

**Figure 9:**
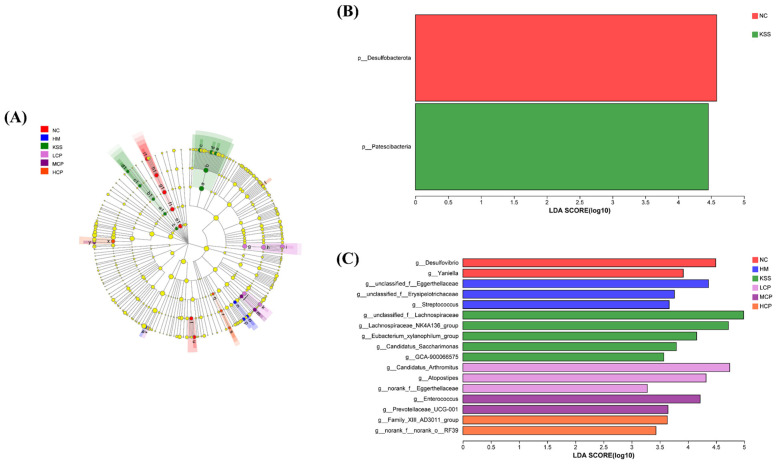
Analysis of evolutionary branching based on LEfSe and the LDA score was 3.0 (**A**) at phylum level (**B**) and genus level (**C**) of the intestinal microbiota. LEfSe was used to identify the enrichment and changes in microbial communities of various populations between groups. NC group: normal control group, HM group: *H. pylori* model group, KSS group: positive group, LCP group: CPAPs with low dose (200 mg/kg·bw), MCP group: CPAPs with medium dose (400 mg/kg·bw), and HCP group: CPAPs with high dose (600 mg/kg·bw).

At the phylum level, *Desulfobacterota* was significantly enriched in the NC group (LDA > 4.5) and *Patescibacteria* was significantly enriched in the KSS group (LDA > 4.5), whereas in other groups, no community was enriched (Figure 9). At the genus level, the KSS group had the most characterized communities, the HM and LCP groups had the second most, and the NC, MCP, and HCP groups had the least. The NC group was significantly enriched in *g_Desulfovibrio* (LDA > 4.5) and *g_Yaniella* (LDA > 4.0). The HM group was significantly enriched in *unclassified_f_Eggerthellaceae* (LDA > 4.0), *unclassified_f_Erysipelotrichaceae*, and *g_Streptococcus* (LDA > 3.5), and these were closely related to intestinal-type changes [45]. The KSS groups *unclassified_f_Lachnospiraceae* (LDA > 5.0), *Lachnospiraceae_NK4A136_group* (LDA > 4.5), *Eubacterium_xylanophilum_group* (LDA > 4.0), *Candidatus_Saccharimonas*, and *GCA-900066575* (LDA > 3.5) were significantly enriched, as were the LCP groups *Candidatus_Arthromitus* (LDA > 4.5), *Atopostipes* (LDA > 4.0), and *norank_f_Eggerthellaceae* (LDA > 3.5). *Enterococcus* (LDA > 4.0) and *Prevotellaceae_UCG-001* (LDA > 3.5) were significantly enriched in the MCP group. The HCP group *g_Family_XIII_AD3011_group* (LDA > 3.5) and *norank_f_norank_o_RF39* (LDA > 3.0) were significantly enriched. The above groups can be considered potential biomarkers for each group. We found that the anti-*H. pylori* effects of CPAPs were closely related to the changes in *Enterococcus* and *Candidatus_Arthromitus*. These results suggest that the CPAP intervention had a positive effect on *H. pylori*-induced intestinal microbiota dysbiosis in mice.

#### 2.3.5. Predictive Analysis of the Intestinal Microbiota Function

To explore the impact of intestinal microbiota function on community shifts, we conducted a functional prediction analysis of the gut microbial community utilizing PICRUST. Referring to the KEGG database, we discovered notable alterations in the function of the intestinal microbial community subsequent to *H. pylori* infection. At level 1 (Table 1), in comparison to the NC group, the HM group exhibited a considerable rise in the relative abundance of genes pertaining to genetic information processing (*p* < 0.05), alongside a substantial reduction in the relative abundance of genes linked to environmental information processing and cellular transformation processes (*p* < 0.05). At level 2 (Table 2), in contrast to the NC group, the HM group demonstrated a marked increase in the relative abundance of genes associated with energy metabolism, translation, replication and repair, nucleotide metabolism, protein folding, and sorting and degradation, as well as infectious disease-causing bacteria (*p* < 0.05). Conversely, there was a significant decrease in the abundance of genes related to membrane transport, cofactor and vitamin metabolism, prokaryotic cellular communities, lipid metabolism, cellular activity, the endocrine system, aging, and transport and catabolism.

The abundance of genes involved in genetic information processing and cellular transformation processes was primarily enhanced by CPAP intervention, aligning with its capability to improve the outcomes of gastric mucosal epithelial cell injury. Compared with the HM group, in terms of human diseases, CPAP intervention significantly decreased the abundance of genes related to cardiovascular diseases (*p* < 0.05). Regarding metabolic pathways, it considerably augmented the abundance of genes involved in lipid metabolism, endocrinology, transport, and catabolism and decreased the abundance of genes related to energy metabolism and nucleotide metabolism (*p* < 0.05). In terms of other pathways, CPAP intervention notably augmented the abundance of genes involved in membrane transport, cellular community—prokaryotes, cellular activity, and aging. Moreover, it significantly diminished the abundance of genes associated with various processes such as translation, replication and repair, folding, and sorting and degradation, as well as those linked to infectious-disease bacteria and transcription (*p* < 0.05).

Alterations in the intestinal microbiota, particularly those possessing specialized functions, will undoubtedly modify the functional landscape of the entire intestinal microbiota. In this study, predicted microbial functions revealed that *H. pylori* infection altered biometabolic pathways. Conversely, CPAPs enhanced biometabolic pathways by adjusting intestinal microbiota composition. This functional prediction implies that CPAPs have the potential to prevent or mitigate metabolic disturbances induced by *H. pylori*.

Collectively, these findings demonstrate that *H. pylori* infection notably disrupted the structure of the gastrointestinal microbiota in mice, resulting in a chaotic response primarily marked by reduced levels of beneficial bacteria and an elevation in harmful bacteria levels. With the intervention of CPAPs, the level of *H. pylori* colonization in the stomach can be significantly decreased, the structural composition of the gastrointestinal microbiota can be enhanced, and the abundance and diversity can be increased, thereby positively regulating the disorders of the gastrointestinal microbiota in mice. In addition, the PICRUST function prediction of intestinal microbiota revealed that CPAPs may prevent or reduce metabolic disorders brought about by *H. pylori*. It is hypothesized that the regulatory impact of CPAPs on gastrointestinal microbiota disorders could be one of the underlying mechanisms through which they counteract *H. pylori*-induced gastric damage.

In addition, according to the maximum dose of 600 mg/kg·bw in this experiment, the human body weight was calculated as 70 kg, which is equivalent to the maximum effective dose of 5.5 g/day for humans.

## 3. Materials and Methods

### 3.1. Materials and Chemicals

*H. pylori* 43504 was obtained from American Type Culture Collect (ATCC, Manassas, VA, USA). Corn gluten meal (CGM) was obtained from FuFeng Development Co., Ltd., (Qiqihar, Heilongjiang, China). Neutrase 0.8 L (21,000 U/mL, determined using the lowry method) was bought from Novo Nordisk (Bagsvaerd, Denmark). Total RNA Kit II R6934 was purchased from Omega Bio-Tek Co., Ltd., (Norcross, GA, USA). PrimeScript™ IV 1st strand cDNA Synthesis Mix 6215 was bought from Takara Biomedical Co., Ltd., (Beijing, China). PowerUp™ SYBR™ Green Master Mix was obtained from Thermo Fisher Scientific (Waltham, MA, USA).

### 3.2. CPAP Preparation

CPAPs were prepared according to our previously reported study [46]. First, CGM was pretreated by extrusion expansion and starch removal. The pretreated CGM was prepared as a suspension at 15% (*w*/*v*), and the pH of the suspension was adjusted to 7.0 with 0.5 M HCl, and the temperature was adjusted to 45 °C. The neutral protease enzyme addition was 400 U/g, and the hydrolysis time was 2 h. After the hydrolysis reaction, the suspension was heated to 95 °C for 10 min and centrifuged at 1000 r/min for 15 min, and the supernatant was freeze-dried (CPAPs). CPAPs are mixtures of peptides.

### 3.3. In Vivo Assay

Forty-eight male KM mice (six weeks old) were purchased from the Changchun Yisi Experimental Animal Research Center (Changchun, China, SCXK (ji)-2018-0007), and were specifically pathogen-free. The animal experiment was approved by the Animal Ethics Committee of the College of Food and Bioengineering, Qiqihar University. The mice were maintained by the Qiqihar University Regulations on Animal Experimentation (Approval No. 2023-002). These mice were allowed to feed and drink freely during the experiment.

**Figure 10:**
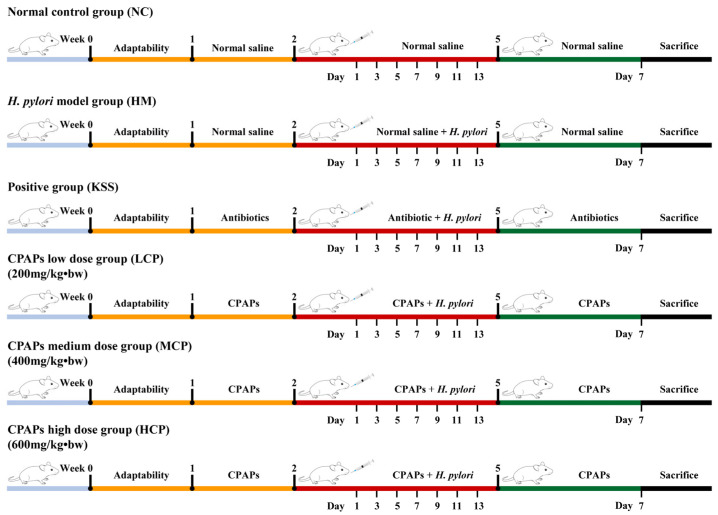
Animal experiment plan. From week 1 to week 2, mice were treated with normal saline, antibiotics, and CPAPs by gavage. From week 3 to week 4, the above substances were continuously administered by gavage, and except for the control group, other groups were given *H. pylori* by gavage every other day. Then we continued the experiment for a week, as in the beginning. After the last dose, the mice were fasted overnight and anesthetized, and the stomachs and intestines of the mice were collected. The samples were frozen at −80 °C for analysis.

The animal experiment plan is shown in Figure 10. The mice were randomly divided into six groups: the normal control group (NC); the *H. pylori* model group (HM); CPAP groups with low (200 mg/kg·bw, LCP), medium (400 mg/kg·bw, MCP), and high (600 mg/kg·bw, HCP) doses; and the positive antibiotic group (14.25 mg/kg amoxicillin + 7.15 mg/kg Clarithromycin + 14.2 mg/kg Metronidazole, KSS). As shown in Figure 10, seven days of adaptive feeding were required. At the beginning of the experiment, the normal and model groups were given normal saline (0.3 mL), the positive control group was given antibiotics (0.3 mL), and the CPAP groups were given different doses of CPAPs. From the third week of the experiment, the groups continued to be given different doses of CPAPs; the normal and model groups were given normal saline, and also started to be given *H. pylori* solution every other day for a total of 7 times (0.4 mL, about 10^9^ cfu/mL). Then we continued the experiment for a week, as in the beginning. After the last dose, the mice were fasted overnight and anesthetized by pentobarbital sodium, and the stomachs and intestines of the mice were collected. The samples were frozen at −80 °C for analysis.

### 3.4. qRT-PCR Assay

Stomach samples from each group of mice were taken for total RNA extraction. Total RNA was extracted using Omega Tissue Total RNA Kit II (R6934) and then reverse-transcribed to cDNA using the PrimeScript™ IV 1st strand cDNA Synthesis Mix 6215 and analyzed in QuantStudio Real-Time Fluorescence Quantitative PCR System (Waltham, MA, USA). For data normalization, β-actin was used as a reference gene; the values of each group were calculated using the 2^−ΔΔCt^ method. Then, we made calculations to obtain the mean value of the NC group; based on the mean value of the NC group, all groups were normalized, and the NC group was normalized to 1. The dispersion of all groups was calculated according to the normalized values of each group, and all results were expressed as means ± standard deviations (SD). The forward and reverse primers for each gene are shown in Table 3. All reactions were run three times.

### 3.5. Analysis of Gastrointestinal Microbiota by 16S rRNA Gene Sequencing

#### 3.5.1. DNA Extraction and Concentration Determination

The samples (the stomachs and intestines) of each group were placed on ice and melted, then DNA extraction reagents were added to be mixed well and extracted evenly and centrifuged at 10,000 r/min for 5 min. The DNA concentration was determined by the NanoDrop2000 Micro Nucleic Acid Analyzer (Waltham, MA, USA), DNA purity was analyzed by 1% agarose gel electrophoresis at the same time, and the brightness of DNA was observed. It was judged whether the extracted DNA met the quality standard.

#### 3.5.2. PCR Amplification and Library Construction

TransGen AP221-02: TransStart Fastpfu DNA Polymerase, 20 μL reaction system was used for PCR amplification assay. In the PCR tube, 4 μL of 5 × FastPfu Buffer, 2 μL of 2.5 mM dNTPs, 0.8 μL of Forward Primer (5 μM), 0.8 μL of Reverse Primer (5 μM), 0.4 μL of FastPfu Polymerase, 0.2 μL of BSA, and 10 ng of template DNA were made up to 20 μL with ddH_2_O. Forward Primer 338F: ACTCCTACGGGGAGGCAGCAG; Reverse Primer 806R: GGACTACHVGGGGTWTCTAAT. PCR reaction parameters: denaturation of the samples at 95 °C for 3 min, the number of cycles was 35, and the single-cycle conditions were as follows—95 °C hold for 30 s, 55 °C hold for 30 s, and 72 °C hold for 45 s. The purity of the PCR products was detected by 2% agarose gel electrophoresis, and the concentration of PCR products was determined by the NanoDrop2000 Micro Nucleic Acid Analyzer (Waltham, MA, USA). PCR libraries were constructed, and DNA fragment sequences were obtained using the Illumina MiSeq sequencing platform (Waltham, MA, USA). The species diversity of each group of samples was analyzed in the Majorbio Cloud platform (www.majorbio.com), including species diversity, community composition, microbiota diversity analysis (α-diversity and β-diversity), microbial community biomarker analysis (LEfSe), and functional predictive analysis (PICRUST and KEGG).

### 3.6. Statistical Analysis

All results were expressed as means ± standard deviations (SD), and statistical comparisons were performed using one-way analysis of variance (ANOVA), and Duncan’s test was used as a post hoc test following ANOVA. The species diversity of each group of samples was analyzed in the Majorbio Cloud platform (www.majorbio.com), including species diversity, community composition, microbiota diversity analysis (α-diversity and β-diversity), microbial community biomarker analysis (LEfSe), and functional predictive analysis (PICRUST and KEGG). Different lowercase letters represent significant differences (*p* < 0.05). SPSS Statistics 26 (SPSS Inc., Chicago, IL, USA) was used to analyze the data.

## 4. Conclusions

In the *H. pylori*-infected mouse model, CPAPs can inhibit the formation of the TLR4/MyD88 complex, which in turn inhibits the activation of the NF-κB signaling pathway, leading to the down-regulation of pro-inflammatory factors and up-regulation of anti-inflammatory factors. Consequently, this alleviates the gastric mucosal damage response induced by *H. pylori*. Additionally, CPAPs effectively enhance the structural composition of the gastrointestinal microbiota by promoting the proliferation of beneficial bacteria and significantly inhibiting the growth of pathogenic bacteria. This promotes the microecological balance of the gastrointestinal tract and plays a crucial role in antagonizing *H. pylori* infection. In the future, we will use multi-omics combined technology to reveal the mechanism of CPAPs against *H. pylori* infection from the perspective of gastrointestinal microorganisms and metabolite analysis.

## Figures and Tables

**Table 1:** Functional prediction analysis of intestinal microbiota (level 1).

KEGG Pathway	NC	HM	KSS	LCP	MCP	HCP
Metabolism	76.61 ± 1.02 a	77.12 ± 0.56 a	75.30 ± 2.32 a	76.82 ± 1.00 a	75.79 ± 0.17 a	76.07 ± 0.23 a
Genetic Information Processing	7.98 ± 0.07 b	8.70 ± 0.42 a	9.33 ± 1.14 ab	8.23 ± 0.04 b	8.15 ± 0.08 b	7.99 ± 0.10 b
Environmental Information Processing	6.68 ± 0.35 ab	6.11 ± 0.42 b	6.31 ± 0.32 b	6.16 ± 0.51 ab	6.75 ± 0.12 b	7.10 ± 0.50 a
Cellular Processes	4.16 ± 0.45 ab	3.62 ± 0.36 ab	4.27 ± 0.65 b	4.08 ± 0.42 a	4.55 ± 0.05 ab	4.35 ± 0.11 ab
Human Diseases	2.96 ± 0.15 a	2.91 ± 0.10 a	3.19 ± 0.47 a	3.07 ± 0.12 a	3.11 ± 0.03 a	2.96 ± 0.06 a
Organismal Systems	1.58 ± 0.03 a	1.50 ± 0.09 a	1.59 ± 0.13 a	1.65 ± 0.06 a	1.65 ± 0.04 a	1.53 ± 0.10 a

Different lowercase letters represent significant differences (*p* < 0.05). NC group: normal control group, HM group: *H. pylori* model group, KSS group: positive group, LCP group: CPAPs with low dose (200 mg/kg·bw), MCP group: CPAPs with medium dose (400 mg/kg·bw), HCP group: CPAPs with high dose (600 mg/kg·bw), and KEGG: Kyoto Encyclopedia of Genes and Genomes.

**Table 2:** Functional prediction analysis of intestinal microbiota (level 2).

KEGG Pathway	NC	HM	KSS	LCP	MCP	HCP
Energy metabolism	4.18 ± 0.20 b	4.96 ± 0.56 a	4.58 ± 0.55 ab	4.18 ± 0.28 b	4.05 ± 0.03 b	4.15 ± 0.11 b
Membrane transport	4.11 ± 0.34 ab	3.54 ± 0.47 b	3.54 ± 0.41 b	3.65 ± 0.50 b	4.18 ± 0.11 ab	4.52 ± 0.43 a
Metabolism of cofactors and vitamins	3.96 ± 0.03 a	3.79 ± 0.02 ab	3.61 ± 0.26 b	3.93 ± 0.10 a	3.87 ± 0.04 a	3.85 ± 0.07 a
Translation	3.36 ± 0.05 b	3.73 ± 0.20 ab	3.99 ± 0.51 a	3.48 ± 0.02 b	3.43 ± 0.04 b	3.34 ± 0.05 b
Replication and repair	2.98 ± 0.06 b	3.24 ± 0.12 ab	3.53 ± 0.51 a	3.08 ± 0.05 b	3.11 ± 0.04 b	3.03 ± 0.04 b
Nucleotide metabolism	2.65 ± 0.05 b	2.76 ± 0.06 a	2.71 ± 0.03 ab	2.76 ± 0.05 a	2.72 ± 0.03 ab	2.68 ± 0.07 ab
Signal transduction	2.57 ± 0.03 b	2.57 ± 0.08 b	2.78 ± 0.12 a	2.51 ± 0.04 b	2.56 ± 0.05 b	2.58 ± 0.07 b
Cellular community—prokaryotes	2.25 ± 0.19 ab	1.94 ± 0.18 c	1.97 ± 0.15 c	2.10 ± 0.11 bc	2.27 ± 0.04 ab	2.39 ± 0.14 a
Lipid metabolism	1.84 ± 0.06 ab	1.72 ± 0.05 b	1.92 ± 0.21 ab	1.96 ± 0.15 a	1.84 ± 0.01 ab	1.76 ± 0.06 ab
Folding, sorting, and degradation	1.47 ± 0.02 c	1.57 ± 0.08 ab	1.59 ± 0.09 a	1.49 ± 0.04 bc	1.45 ± 0.01 c	1.45 ± 0.02 c
Metabolism of other amino acids	1.24 ± 0.08 ab	1.27 ± 0.05 ab	1.38 ± 0.12 a	1.31 ± 0.13 ab	1.20 ± 0.00 b	1.18 ± 0.02 b
Cell motility	0.98 ± 0.24 ab	0.78 ± 0.13 b	1.34 ± 0.50 a	0.93 ± 0.32 ab	1.25 ± 0.08 ab	1.03 ± 0.09 ab
Infectious disease: bacterial	0.69 ± 0.01 b	0.73 ± 0.04 ab	0.91 ± 0.26 a	0.69 ± 0.03 b	0.71 ± 0.03 b	0.64 ± 0.02 b
Endocrine system	0.57 ± 0.02 ab	0.51 ± 0.07 bc	0.47 ± 0.06 c	0.60 ± 0.04 a	0.59 ± 0.02 ab	0.56 ± 0.04 ab
Aging	0.28 ± 0.05 ab	0.25 ± 0.01 ab	0.30 ± 0.05 a	0.30 ± 0.04 ab	0.27 ± 0.01 ab	0.24 ± 0.00 b
Environmental adaptation	0.21 ± 0.01 ab	0.20 ± 0.01 ab	0.26 ± 0.06 a	0.23 ± 0.01 ab	0.23 ± 0.01 ab	0.20 ± 0.02 b
Transport and catabolism	0.19 ± 0.02 ab	0.17 ± 0.03 b	0.19 ± 0.06 ab	0.27 ± 0.05 a	0.24 ± 0.03 ab	0.19 ± 0.06 ab
Transcription	0.17 ± 0.01 b	0.19 ± 0.01 ab	0.21 ± 0.04 a	0.17 ± 0.01 b	0.16 ± 0.01 b	0.16 ± 0.00 b
Cardiovascular disease	0.15 ± 0.01 b	0.17 ± 0.01 a	0.14 ± 0.02 ab	0.15 ± 0.01 ab	0.16 ± 0.00 ab	0.16 ± 0.01 ab
Neurodegenerative disease	0.12 ± 0.01 a	0.11 ± 0.00 ab	0.13 ± 0.00 a	0.14 ± 0.02 a	0.12 ± 0.00 ab	0.10 ± 0.01 b

Different lowercase letters represent significant differences (*p* < 0.05). NC group: normal control group, HM group: *H. pylori* model group, KSS group: positive group, LCP group: CPAPs with low dose (200 mg/kg·bw), MCP group: CPAPs with medium dose (400 mg/kg·bw), HCP group: CPAPs with high dose (600 mg/kg·bw), and KEGG: Kyoto Encyclopedia of Genes and Genomes.

**Table 3:** Primer information of qRT-PCR experiment.

Gene	Forward	Reverse	Gene ID
β-actin	ATCCGGACCCTCCATTGTC	AGCCATGCCAATCTCGTCTT	11461
TGF-β	ACCTCGACACCGACTACTGCTT	ACTCTTGCGGAAGTCGATGT	21812
IL-10	CGGGAGCTGAGGGTGAA	GTGAAGAAGCGGTGACAGC	16153
TLR4	GTTCCTGCTGAAATCCCAAA	TATGGATGTGGCACCTTGAA	21898
MyD88	TGATGCCTTCATCTGCTACTG	TCCCTCCGACACCTTCTTTCT	17874
NF-κB	GTGTGAAGAAACGGGAACTG	GGCACGGTTGTCATAGATGG	18033

## Data Availability

Data will be made available on request.
